# Sex and Age Differences in Decision-Making Under Risk by Wild Balinese Long-Tailed Macaques (*Macaca fascicularis fascicularis*): A Field Experimental Study

**DOI:** 10.3390/ani16040617

**Published:** 2026-02-15

**Authors:** Caleb Bunselmeyer, Noëlle Gunst, I Nengah Wandia, Robert J. Williams, Elsa Addessi, Jean-Baptiste Leca

**Affiliations:** 1 Department of Psychology, Faculty of Arts & Science, University of Lethbridge, Lethbridge, AB T1K3M4, Canada; bunselmeyer@uleth.ca (C.B.); noelle.gunstleca@uleth.ca (N.G.); 2 Primate Research Center, Faculty of Veterinary Medicine, Udayana University, Denpasar, Bali 80361, Indonesia; wandia@unud.ac.id; 3 Faculty of Health Sciences, University of Lethbridge, Lethbridge, AB T1K3M4, Canada; robert.williams@uleth.ca; 4 CNR, Istituto di Scienze e Tecnologie della Cognizione, 00197 Rome, Italy; 5 School of Natural and Engineering Sciences, National Institute of Advanced Studies, Bangalore 560012, India

**Keywords:** risky decision-making, risk preference, win–stay strategy, foraging choice task, field experiment, long-tailed macaques

## Abstract

This study examines risky decision-making in a free-ranging population of Balinese long-tailed macaques (*Macaca fascicularis fascicularis*), addressing gaps in research that have largely focused on captive primates and have rarely considered individual differences by age and sex. Thirty-three macaques of different age–sex classes were tested using a choice task contrasting a guaranteed small reward with a probabilistic larger reward. At the group level, macaques showed no preference for safe or risky options. However, substantial individual variation emerged: some individuals were risk-prone, others risk-averse, and many indifferent. Notably, age and sex interacted in shaping risk preferences. Among males, adults and juveniles were more risk-prone than younger adults, whereas among females, adults were more risk-prone than juveniles. Juveniles also displayed outcome-dependent flexibility, choosing the risky option more often after a previous successful risky choice, consistent with a win–stay strategy. Like in rodents, this pattern may reflect adaptive learning during developmental transitions. Importantly, the observed behavioral differences were not due to misunderstanding of the task, as macaques reliably chose the larger option when outcomes were visible. This pronounced individual variability in primate risk preferences underscore the importance of considering demographic factors when characterizing species-typical risk preferences.

## Simple Summary

Most past research on risk preferences in primates focused on captive animals and neglected individual differences. We studied how 33 wild Balinese long-tailed macaques of different ages and sexes make risky choices. The animals were given a choice between a small reward they would always get and a larger reward they would get only sometimes. Overall, the macaques did not prefer either the safe or the risky option. However, individuals differed greatly. Some macaques often chose the risky option, others preferred the safe one, and many showed no clear preference. These differences were strongly linked to age, sex, and what happened in previous trials. Age and sex worked together in shaping risk preferences. Among males, juveniles and older adults were more risk-prone than younger adults. Among females, adults were more risk-prone than juveniles. Juveniles were also flexible in their choices, tending to repeat a risky choice after it paid off, a pattern known as “win–stay, lose–shift”. This may help young animals learn during development. Importantly, these differences were not due to confusion, since the macaques made correct choices when outcomes were clearly visible. Our findings highlight strong individual variation in primate risk preferences shaped by demographic factors.

## 1. Introduction

Risk, defined as variability in the rate of gains [1], pervades the daily life of wild animals. Due to the unpredictable nature of their environments, animals face risks on a daily basis across diverse contexts such as foraging, seeking mating opportunities, and avoiding predators [2]. In decisions involving risk, individuals typically choose between a safe option (offering a small but certain reward) and a risky alternative (offering a potentially larger yet uncertain reward), with known probabilities assigned to each outcome [3,4]. Numerous comparative studies have examined risk preferences across various primate species with different feeding ecologies. Species usually exploiting more variable food sources, including rhesus macaques (*Macaca mulatta*), capuchin monkeys (*Sapajus* spp.), chimpanzees (*Pan troglodytes*), and orangutans (*Pongo abelii*), exhibited a risk-prone attitude when faced with probabilistic payoff variations. Conversely, species reliant on more stable resources or inhabiting harsh environments, such as bonobos (*Pan paniscus*), gorillas (*Gorilla gorilla*), and lemurs (*Lemur catta*, *Eulemur mongoz*, *Varecia rubra*) displayed risk-averse tendencies [5,6]. Nonetheless, more recent research revealed that this overarching pattern is not always consistent. Rhesus macaques, capuchin monkeys, and chimpanzees exhibit varying risk preferences—from risk-seeking to risk-averse—depending on the task and contextual factors [4,7,8,9,10,11,12]. This variability suggests that observed inter-specific differences in risk preferences might instead reflect interindividual variation within species.

Sex and age represent two relevant demographic factors that may shape risk preferences, though experimental evidence is still limited. Regarding sex differences, several studies report that male chimpanzees and capuchin monkeys exhibit greater risk tolerance compared to females. In the wild, male chimpanzees were more frequently observed hunting colobus monkeys (*Procolobus* spp.), a potentially hazardous behavior that only sometimes results in success [5,13]. Similarly, in a population of sanctuary-living chimpanzees tested in a risky-choice task, males demonstrated greater risk preferences than females [10]. This pattern is echoed in captive male capuchin monkeys tested in an analogous task [14]. Nevertheless, other studies have not found significant sex differences in risk preference within these species [3,4,15,16], with failures not evidently attributable to sample size limitations [5].

Regarding age differences, in sanctuary-living chimpanzees tested in a risky-choice task, adolescent and young adults exhibited greater risk proneness compared to older adults [10,17]. This developmental pattern, characterized by higher risk proneness during adolescence followed by a more conservative approach during adulthood, closely mirrors the well-documented trend observed in humans [18]. This parallel suggests that heightened risk proneness during adolescence may be linked to a higher necessity of exploring the environment, competing for social status, and/or dispersing from the natal group. However, a study on probability inference in rhesus macaques [19] reported comparable risk preferences between younger and older individuals. A more nuanced pattern emerged from a recent study on capuchin monkeys [12], revealing a three-way interaction among task contingencies, age, and past experience. Specifically, older monkeys demonstrated a higher propensity to take risks compared to younger ones when learning outcome probabilities through experience, with a lower age-related risk proneness in individuals with greater prior experience in risky-choice tasks.

Risk preferences have been extensively investigated in captive non-human primates through paradigms involving learned associations, intuitive outcomes and exchange tasks, respectively reviewed in [5]. In the first paradigm, subjects first underwent a training phase during which they learned the expected values of a certain option and a probabilistic alternative, followed by repeated choices between these options. The certain option yielded a constant payoff, whereas the risky option provided a reward that varied probabilistically in quantity or quality [3,4]. In the second paradigm, subjects could choose between an upside-down cup concealing a small certain reward, and multiple upside-down cups, only one of which hid a larger reward with a given probability [16,20]. In the third paradigm, subjects exchanged a lower-value food item with a human experimenter to obtain a larger reward under probabilistic conditions [21,22]. These controlled experimental approaches provided valuable insights, yet it remains unclear whether their findings extend to wild populations. Previous research has extensively examined probabilistic inferences [19], foraging decision-making [23], and predatory risk perception [24] in wild non-human primates, but no studies to date have investigated decision-making under risk in wild populations using paradigms comparable to those applied in captive settings.

We tested a sample of 33 Balinese long-tailed macaques (*Macaca fascicularis fascicularis*) from a free-ranging, urban-dwelling, habituated, provisioned population with a diverse age and sex composition, employing a risky-choice task. Subjects were tested opportunistically by presenting each individual with a series of choices between a safe option (a single upside-down colored cup consistently concealing a single food item) and a risky option (four upside-down colored cups, one of which concealed four food items). To confirm that choices in the main task reflected an understanding of the contingencies rather than chance, control trials were conducted in which macaques observed the baiting process, making clear which risky cup contained the larger reward.

Long-tailed macaques are a good primate model for studying decision-making under risk because they combine several advantages that are rare to find in another species. First, in terms of behavioral ecology, they are opportunistic foragers, frequently facing unpredictable situations in the wild that require balancing risk against potential payoff [25,26,27]. Second, in terms of cognitive flexibility, they can adapt rapidly to a changing environment [28]; they perform well in experimental tasks involving categorization [29]; they show varying degrees of inequity aversion with different cost–benefit ratios [30]; and they display relatively long waiting abilities in exchange task [22]. Third, they show temperamental characteristics and stable personality variations that are relevant to investigating their risk preferences [31,32].

Because our study population was provisioned with food daily, it was buffered from major temporal and spatial variability in food abundance. Still, studies showed that long-tailed macaque groups relying on food provided by humans also change their movement and feeding patterns depending on human presence and activities (e.g., food offerings during religious ceremonies, visitors bringing food to the site), which could be considered a proxy for unpredictable food availability [33]. Moreover, all our studied subjects routinely and spontaneously engage in token-mediated bartering interactions with humans that typically involve the following three steps: (1) a monkey steals a valued object from a visitor to the temple (e.g., glasses, hats, jewelry); (2) the monkey waits for one of the temple staff to provide a food offering in exchange for the object; (3) when the monkey deems that the food offering is sufficient, it provides the object to the temple staff who then returns it to the visitor [34]. Such unusual behavioral sequences involve risk-sensitive decisions pertaining to food procurement.

According to the foraging hypothesis, which posits that ecological pressures are crucial drivers of cognitive evolution in primates [6], the ecological and behavioral characteristics of long-tailed macaques would lead us to expect a risk-prone attitude when individuals choose between a safe option and a risky option with the same expected value. However, to our knowledge, the only study that has investigated the risk preference of long-tailed macaques reported a neutral risk attitude in two captive individuals tested using a repeated information-sampling task, in which they could interact with an automatically operating food dispenser [35]. Consequently, precise predictions based on the existing literature are difficult to formulate, and the present study should therefore be regarded as exploratory. Nevertheless, by drawing on a larger sample of wild individuals, the findings of this study will contribute to further elucidating the factors influencing decision-making under risk within the Primate order.

## 2. Materials and Methods

### 2.1. Ethical Note

All subjects were well habituated to humans and tested opportunistically. Macaques could participate voluntarily by approaching the experimenter and stop participating at any time by just moving away. The research was non-invasive, and all tests were motivated by positive reinforcement. The study was conducted in accordance with the Indonesian Ministry of Research and Technology (research permits: 253/SIP/IV/FR/10/2022 and 325/SIP/IV/FR/5/2024), the Provincial Government of Bali and the local district authorities. It was approved by the institutional Animal Welfare Committee of the University of Lethbridge (Protocol number 2201).

### 2.2. Study Population and Site

We studied a population of free-ranging, urban-dwelling, habituated, and provisioned Balinese long-tailed macaques living within and around the Uluwatu Temple (8°49′ S, 115°05′ E), located in a dry agricultural landscape of southern Bali, Indonesia. This is a Hindu temple complex used by Balinese communities for daily religious ceremonies. It is also one of the most famous tourist spots on the island, visited by 1.5 million tourists in 2015 [36]. A population of long-tailed macaques has lived in this anthropogenic habitat for decades [37]. At the time of the study, the population totaled approximately 400 individuals and was composed of five neighboring groups with overlapping home ranges [36]. The monkeys have been provisioned by the temple staff sporadically since 1999, and regularly since 2010 with various fruits and vegetables widely distributed across the site [38]. They also receive fruits and peanuts from tourists.

### 2.3. Subjects

We tested 33 individually identified subjects, including six adult females (>4 years old), seven adult males (>6 years old), three subadult females (3–4 years old), eight subadult males (4–6 years old), two juvenile females (1–3 years old), and seven juvenile males (1–4 years old). Even though the exact age and genealogy of studied subjects were not known, we determined age–sex classes by visual assessment of body size and proportion, dental development, and sexual maturity features (see [39] for the categorization of age and sex classes in Balinese long-tailed macaques). Individual identification was achieved by using a combination of natural physical markers, such as facial features, body size and proportions, fur coloration and patterns, as well as long-lasting scars, wounds, and deformities.

### 2.4. General Procedure

Testing was carried out during two field seasons (November 2022–January 2023 and June–August 2024) and occurred between 8:00 a.m. and 6:00 p.m. The subjects were tested opportunistically whenever the experimenter encountered one or more individuals and at least one individual was willing to approach the experimental apparatus. Throughout the study, the food item unit used as a reward was one slice of sweet banana, locally known as Pisang raja. A conditioning phase preceded experimental trials for macaques that had not interacted with experimental apparatus, wherein a food item was placed on the board in view, and the macaque was allowed to freely approach the board and take the food item (see paragraph 2.5 for a description of the experimental apparatus).

The study consisted of an Experimental phase and a Control phase. In both phases, one experimenter positioned on the ground and in front of an approaching macaque a wooden board on which there were (a) a single upside-down colored cup always covering one food item and (b) four upside-down differently colored cups, one of which covered four food items. Then, the experimenter waited until the macaque chose one of the cups by either touching or lifting it. During the choice procedure, the experimenter did not look at any cup to prevent unintentional cues. A second experimenter video recorded all trials.

### 2.5. Experimental Apparatus

In both Experimental and Control phases, the apparatus was a wooden board (measuring 60 × 10 cm). Attached to the board was a layer of poster board on which five dowel rods (measuring 30 cm) were evenly spaced across the board. Upside-down cups were positioned on the dowel rods to allow the macaques to lift them up to obtain the food item. The cups were all attached to the dowel rod (measuring 60 cm) by dental floss to allow the experimenter to lift all five cups at the beginning and end of each trial. The board was placed on the ground, in front of the experimental subject. In both phases, there were a single upside-down, black-striped light blue cup always covering one food item and four upside-down differently colored cups, one of which covering four food items. Due to material availability in Bali, in the 2022 field season we used four green cups, whereas in the 2024 field season we used four red cups. Each cup measured 8.9 cm in diameter and 12.7 cm high. To introduce uncertainty in the Experimental phase, the experimenter concealed baiting from the experimental subject’s view by means of an opaque screen. In the 2022 field season, cardboard was used for the screen and in the 2024 field season a wooden screen was used.

### 2.6. Experimental Phase

In the Experimental phase, macaques were presented with a series of choices between a safe option (a single upside-down, black-striped light blue cup always covering one food item) and a risky option (four upside-down green or red cups, one of which covering four food items). The safe option was consistently placed at either end of the row, with its order counterbalanced across trials. The expected value (EV) was the same for both options. For the risky option, there was a 0.25 probability (P) of receiving four food items (V). Thus, the EV was 0.25 × 4 = 1, which matched the EV of the safe option, yielding one food item with a probability of 1.

Before baiting the cups, the experimenter lifted the five cups to show the macaque each upside-down cup was empty. The experimenter then showed the macaque subject the potential food amounts associated with each option (one and four food items, respectively) in their open hands. After the subject had looked at both food amounts, the experimenter placed those under the respective upside-down cups. The experimenter placed the single food item under the cup representing the safe option in full view of the subject, whereas they concealed the baiting procedure of the four food items, which were placed under one of the four cups, by means of an opaque wooden or cardboard screen. The hiding position of the four food items was pseudo-randomized across trials. Once the screen covering the risky option was removed, the subject could select their preferred cup by touching or lifting it. After each rewarded choice, there were two possible scenarios. In the first one (which occurred most of the time), the subject took the food reward off the board after lifting the cup. In the second scenario, if the subject did not take the food reward, the experimenter provided it. The experimenter also revealed the content of the non-chosen option, allowing the subjects to see where the food items were located. There was an inter-trial interval of about 15 s. Supplementary Material Video S1 shows a typical experimental session during which the subject chose the risky option, and Supplementary Materials Video S2 shows a typical experimental session during which the subject chose the safe option (https://osf.io/yh8mr/files/95umw, 10 February 2026).

We usually carried out one experimental session per day. Most subjects (*N* = 27) participated in one session, three subjects in two sessions, two subjects in three sessions, and one subject in five sessions. Each session had an average of 11.93 ± 0.21 trials (minimum 9 trials–maximum 13 trials). Further details are reported in Table 1.

### 2.7. Control Phase

The Control phase aimed to assess whether macaques consistently chose the cups covering the largest food option when they could observe the baiting procedure. This ensured that their choices in the Experimental phase were influenced by the uncertainty of obtaining the better payoff rather than by other factors. The only difference between the Control phase and the Experimental phase was that, in the Control phase, the experimenter baited also the risky option in full view of the subject. This ensured that macaques could see, before making their choice, under which cup the four food items were placed.

We carried out the Control phase after the Experimental phase on 24 out of the 33 macaques, which participated in the Experimental phase during the 2024 field season. Most subjects (*N* = 22) participated in one session, one subject in two sessions, and one subject in three sessions. Each session included a single choice between the single upside-down, black-striped light blue cup covering one food item and the four upside-down red cups covering four food items.

### 2.8. Data Analysis

To assess whether macaques significantly preferred either option above chance level, we employed the Binomial test at the individual level and the single-sample Wilcoxon signed-ranks test at the population level.

Next, to evaluate whether sex, age, trial number, and previous trial outcome (zero, one, or four food items) were related to risky choices, we implemented a random-effects logistic regression model. The identity of each subject was included as a random effect. Interactions were analyzed using the Wald test; non-significant interactions were removed from the model before repeating the analysis. The significance level was set at *p* < 0.05. We conducted all analyses using Stata 17.0.

## 3. Results

### 3.1. Experimental Phase

The average proportion of choices for the risky option did not differ significantly from random choice (mean ± SE: 0.41 ± 0.29, z = −1.662, *p* = 0.097). As reported in Table 1, at the individual level, six macaques were risk-prone, 15 macaques were indifferent between options, and 12 macaques were risk-averse. Specifically, two adult females were risk-prone and four were indifferent (overall prone: z = 3.481, *p* exact < 0.001); one adult male was risk-prone, three were indifferent, and three were averse (overall indifferent: z = −1.949, *p* exact = 0.064); two subadult females were risk-prone, one was averse (overall indifferent: z = 1.333, *p* = 0.243); two subadult males were indifferent and six were averse (overall averse: z = −8.803, *p* exact < 0.001); one juvenile female was averse and the other one was indifferent (overall averse: z = −2.667, *p* exact = 0.011); one juvenile male was risk-prone, five were indifferent, one was averse (overall indifferent: z = 0.863, *p* exact = 0.450).

**Figure 1:**
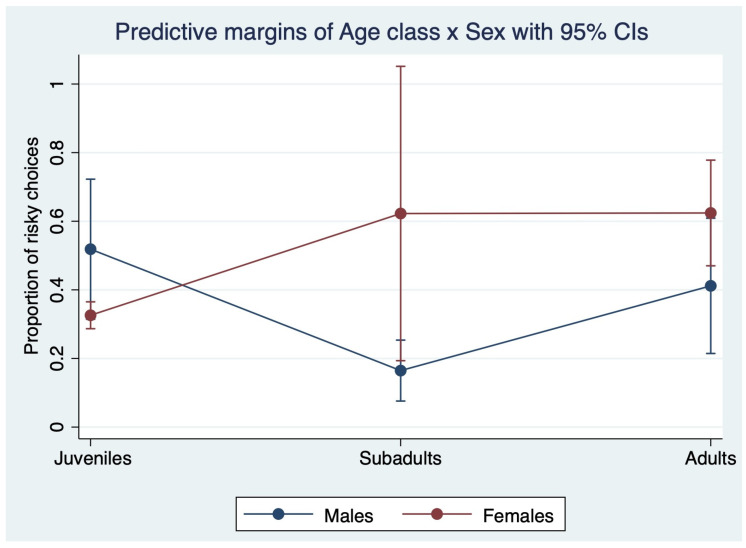
The graph reports marginal means and confidence intervals for the dependent variable “risky choices”, and depicts the significant interaction between age (juveniles, subadults, adults) and sex (males, females).

**Figure 2:**
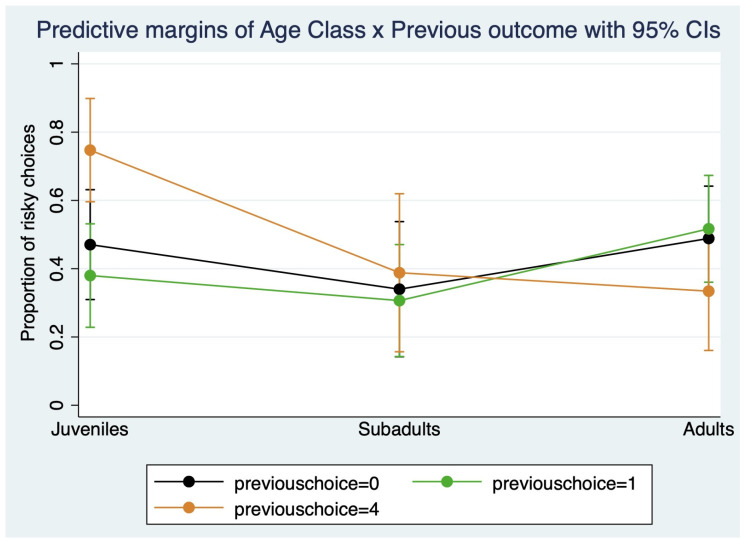
The graph reports marginal means and confidence intervals for the dependent variable “risky choices”, and depicts the significant interaction between age (juveniles, subadults, adults) and outcome of the previous choice. (0 = risky with unfavorable outcome, 1 = safe option, 4 = risky with favorable outcome).

As shown in Figure 1 and Figure 2, there were significant interactions between age and sex (Chi square = 11.23, *p* = 0.004, df = 2), and between age and previous trial outcome (Chi square = 15.91, *p* = 0.003, df = 4); however, the triple interaction between age, sex and previous trial outcome was not significant (Chi square = 2.82, *p* = 0.588, df = 4).

Among males, adults (marginal mean ± SE: 0.41 ± 0.10) and juveniles (0.52 ± 0.10) showed a significantly higher risk preference than subadults (0.16 ± 0.04) (adults vs. subadults: coeff. = 1.495, z = 2.39, *p* = 0.017; juveniles vs. subadults: coeff. = 2.031, z = 3.23, *p* = 0.001); among females, adults (0.62 ± 0.08) were significantly more risk-prone than juveniles (0.33 ± 0.02) (coeff. = 1.484, z = 3.53, *p* < 0.001).

Juveniles chose the risky option significantly more after having previously chosen a risky option producing a four-food-item outcome (0.75 ± 0.08) compared to a risky option with no food outcome (0.47 ± 0.08) or a safe option (0.38 ± 0.08) (risky with four-food-item outcome vs. no food outcome: coeff. = 1.495, z = 3.01, *p* = 0.003; risky with four-food-item outcome vs. safe: coeff. = 1.957, z = 3.09, *p* = 0.002). There were no other statistically significant comparisons.

### 3.2. Control Phase

When the subjects could observe the baiting procedure, and thus were aware of which risky cup concealed the larger food amount, they selected the cup covering four food items in 0.81 ± 0.08 of the trials, on average.

## 4. Discussion

In the present study, we tested a sample of 33 Balinese long-tailed macaques from a free-ranging, urban-dwelling, habituated, provisioned population in a risky-choice task. Overall, macaques exhibited indifference between the safe option (yielding one food item) and the risky option (corresponding to a 25% chance of receiving four items). However, we found important interindividual differences related to age–sex interactions. Specifically, adult and juvenile males were more risk-prone than younger adult males, and adult females were more risk-prone than juvenile females. Additionally, regardless of sex, juvenile macaques were sensitive to the outcome of their prior choices compared to adults. More specifically, they showed a “win–stay” strategy, by selecting the risky option more often after having previously chosen a “winning” risky option compared to a “losing” risky option or a safe option.

Macaques’ indifference between the safe and the risky options may be due either to a lack of understanding of the contingencies of the task or to economic rationality, as the two options had the same expected value. The control trials performed on a subset of subjects, in which macaques yielded 81% accuracy in identifying rewarded cups when they could witness the baiting procedure, dismissed the possibility that they did not understand the task. Thus, it seems more likely that, on average, this population of long-tailed macaques showed genuine risk neutrality. Notably, at the individual level, indifference between options was the pattern most frequently observed, notwithstanding striking interindividual differences. To our knowledge, the only other study investigating attitude towards risk in two long-tailed macaques tested in a repeated information-sampling task showed a neutral risk preference [35].

However, the risk neutrality of long-tailed macaques contrasts with the risk-seeking behavior observed in most of the captive studies on rhesus macaques carried out so far [8,9,40]; but see [7,41]. Several factors may account for the different risk preferences of the two species. First, rhesus and long-tailed macaques, while phylogenetically closely related [42], show some differences in social tolerance and temperament: rhesus macaques are slightly more despotic than long-tailed macaques [43,44], and rather aggressive and unsociable towards humans, whereas long-tailed macaques are more cautious and fearful [32]. Additionally, when tested in a battery of inhibitory control tasks, rhesus macaques showed worse performance than long-tailed macaques ([45]; see also [46] for delay of gratification in long-tailed macaques and [47] for a critical discussion), which may explain their higher risk preferences, although there are mixed results regarding the association between risk preferences and delay preferences in both human and non-human primates [48,49]. Second, captive rhesus macaques are usually presented with repeated situations whose contingencies must be learned by experience, which produce a systematic bias towards risk-proneness [50,51]. In contrast, we tested wild long-tailed macaques with a risky-choice paradigm in which the probability distribution was visually described by the four cups, only one of which contained the larger reward. Third, captive rhesus macaques tested in risky-choice tasks are usually food-deprived to increase their motivation, and thus in a low energetic state, leading to higher risk proneness according to risk-sensitivity theory [1]. By comparison, the long-tailed macaques sampled in our study were not only free to forage, but they were also provisioned, and this may have increased their energetic state, leading them to be overall indifferent between options rather than risk prone as captive rhesus macaques. Finally, whereas captive studies are usually carried out on a few individuals, here we tested a rather large population of macaques and, despite the overall risk neutrality, we found notable interindividual differences. This variability suggests that the observed interspecific differences in risk preference may at least in part reflect interindividual variation within species.

In the present study, adult and juvenile males showed a more pronounced risk-seeking attitude than younger adult males, and adult females were more risk-prone than juvenile females. These findings are not consistent with the few reports on the effect of age on risk attitude in non-human primates, and particularly studies in chimpanzees showing that adolescents and young adults exhibited greater risk proneness than older adults [10,17]. There may be different motives beyond the higher risk preference of juvenile and adult males, which were overall risk neutral, compared to younger adult males, which were risk-averse. Juveniles’ choices may be guided by impulsive tendencies and low awareness of the potentially negative consequences of their behavior [52]. In contrast, adult males may be bolder than younger males because of their generally higher explorative and competitive attitude in social, sexual, and foraging contexts [53,54]. Similarly, the higher risk proneness of adult females, as compared to juvenile females, contrasts with the typical females’ risk-aversion behavior reported in the literature [5]. There are at least two related explanations for this deviation from the usual pattern. First, adult females’ reproductive burden is likely to increase their energy needs compared to immature females [55]. Second, according to the abundance/risk hypothesis [56], food provisioning of our studied macaque population may have favored risk proneness among adult females. Interindividual differences were, however, conspicuous, and it cannot be excluded that a few individuals have driven the group effect.

Finally, regardless of sex, juvenile macaques were sensitive to the outcome of their prior choices, showing a short-term “win–stay” strategy, i.e., selecting the risky option more often after having just chosen, in the previous trial, a “winning” risky option rather than a “losing” risky option or a safe option. Win–stay/lose–shift-like heuristics are often observed in non-human primates and other animals tested in risky-choice tasks [57]; reviewed in [39]. However, this is the first study, to our knowledge, in which juveniles weighted their recent positive outcomes more so than older individuals, possibly because the rewarding risky choice reinforced their exploratory tendencies. Although the risky-choice task was novel for all macaques, the different past experiences with ecological contingencies of juvenile and older individuals may have played a role, leading older individuals to adopt choice strategies less influenced by recent positive outcomes. This hypothesis is supported by the observation that the win–stay/lose–shift heuristic is not consistent across different species and populations of the same species. Some studies identified sensitivity to previous choice outcomes in rhesus macaques tested with risky-choice tasks [58,59], whereas others reported interindividual differences [60]. Additionally, when tested with the same paradigm, bonobos modulated their risky choices based on previous outcome, whereas chimpanzees did not [57] (see also [61] for similar negative findings in another risky-choice paradigm). Similarly, two colonies of tufted capuchin monkeys exposed to slightly different feeding schedules were differently sensitive to previous risky-choice outcomes [3,14]. Taken together, these findings indicate that reliance on recent positive outcomes varies across primate species and populations, likely reflecting differences in life history and ecological features.

While the present study offers important insights into risky decision-making in non-human primates, a specific methodological limitation should be acknowledged. Because our data collection was based on the spontaneous and voluntary participation of free-ranging individuals in the proposed choice task, our results may have been biased via a possible self-selection of study subjects. Indeed, several field experimental studies conducted in wild primates have shown that personality traits lead to systematic interindividual differences in task engagement and learning in natural environments. Specifically, bolder, more active, and more exploratory individuals are more likely to approach, interact with, and solve human-induced problems than their shyer and generally more inhibited counterparts [62,63,64]. Even though this issue is inherent to most field experiments, we cannot rule out an oversampling of bolder monkeys in our study, which could have impacted the generalizability of our results on risk preferences. For example, such a self-selection process could explain the preponderance of male subjects among juveniles and subadults (Table 1), that is, age categories in which sex differences in boldness and risk-taking are often more pronounced than in older individuals, particularly in macaques [65,66].

## 5. Conclusions

In conclusion, this study provided the first examination of risk preferences in free-ranging Balinese long-tailed macaques, extending laboratory paradigms on risky choices to field settings, and thus bridging a critical gap in achieving a more complete understanding of risk attitude in a more ecologically valid setting. Our findings revealed that this urban-dwelling population exhibited overall risk neutrality in a descriptive risky-choice task. This contrasts with the risk-prone tendencies of captive rhesus macaques and highlights the potential influence of ecological context, provisioning, and species-specific features on decision-making under risk. Interindividual variability driven by age–sex interactions underscores that risk preferences are potentially affected by a complex interplay of factors, such as developmental stage, reproductive demands, and experience, which all shape risk attitude, and highlight the importance of considering demographic factors when characterizing species-typical risk preferences. These findings call for future research on decision-making under risk in a longitudinal perspective in a wide variety of captive and free-ranging non-human primate populations, possibly experiencing different degrees of food availability, to provide a framework for reconciling inter- and intra-specific variability in non-human primate attitude towards risk.

## Figures and Tables

**Table 1:** Demographic information on the experimental subjects (AF = adult females, AM = adult males, JF = juvenile females, JM = juvenile males, SAF = subadult females, SAM = subadult males), number of experimental sessions, experimental trials, risky choices (four-food-item risky choices are reported in parenthesis), safe choices, and risk preference categorization (according to the Binomial test * *p* < 0.05, ** *p* < 0.01, *** *p* < 0.001).

ID	Age Class	Sex	Group	Field Season	Experimental Sessions	Experimental Trials	Risky Choices	Safe Choices	Risk Preferences
AF1	Adult	F	Gading	2024	3	36	32(8)	4	Prone ***
AF2	Adult	F	Gading	2022/2024	2	27	14(5)	13	Indifferent
AF3	Adult	F	Gading	2024	1	12	7(2)	5	Indifferent
AF4	Adult	F	Reting	2024	1	13	5(1)	8	Indifferent
AF5	Adult	F	Gading	2024	1	10	4(1)	6	Indifferent
AF6	Adult	F	Gading	2022	1	15	13(6)	2	Prone **
AM1	Adult	M	Pongya	2024	1	12	4(2)	8	Indifferent
AM2	Adult	M	Reting	2024	1	12	2(1)	10	Averse *
AM3	Adult	M	Reting	2024	1	12	10(5)	2	Prone *
AM4	Adult	M	Melem	2024	1	12	7(4)	5	Indifferent
AM5	Adult	M	Gading	2024	2	22	12(2)	10	Indifferent
AM6	Adult	M	Gading	2024	1	12	2(0)	10	Averse *
AM7	Adult	M	Reting	2024	1	13	1(0)	12	Averse **
JF1	Juvenile	F	Gading	2024	2	24	7(1)	17	Averse *
JF2	Juvenile	F	Gading	2024	1	12	3(0)	9	Indifferent
JM1	Juvenile	M	Gading	2024	1	12	9(1)	3	Indifferent
JM2	Juvenile	M	Gading	2024	1	12	3(1)	9	Indifferent
JM3	Juvenile	M	Gading	2024	1	12	5(1)	8	Indifferent
JM4	Juvenile	M	Gading	2024	1	12	11(3)	1	Prone **
JM5	Juvenile	M	Gading	2024	1	12	7(2)	5	Indifferent
JM6	Juvenile	M	Gading	2022	1	17	11(2)	6	Indifferent
JM7	Juvenile	M	Pongya	2022	1	9	1(0)	8	Averse *
SAF1	Subadult	F	Reting	2024	1	12	1(1)	11	Averse **
SAF2	Subadult	F	Gading	2024	1	11	10(2)	1	Prone *
SAF3	Subadult	F	Reting	2024	1	13	11(3)	2	Prone **
SAM1	Subadult	M	Gading	2024	3	34	6(2)	28	Averse ***
SAM2	Subadult	M	Gading	2024	1	12	1(1)	11	Averse **
SAM3	Subadult	M	Gading	2024	1	12	0	12	Averse ***
SAM4	Subadult	M	Gading	2022	1	9	4(2)	5	Indifferent
SAM5	Subadult	M	Reting	2024	1	12	3(1)	9	Indifferent
SAM6	Subadult	M	Gading	2024	5	58	9(2)	49	Averse ***
SAM7	Subadult	M	Gading	2024	1	11	1(1)	12	Averse **
SAM8	Subadult	M	Gading	2024	1	11	0	11	Averse ***

## Data Availability

The original data presented in the study are openly available in OSF at https://osf.io/yh8mr/files/yr526 (accessed on 10 February 2026).

## Supplementary Materials

The following supporting information can be downloaded at: https://osf.io/yh8mr/files/95umw (accessed on 10 February 2026), Video S1: Choice of the risky option; Video S2: Choice of the safe option.

## References

[1] Kacelnik A., Bateson M. (1996). Risky theories—The effects of variance on foraging decisions. Am. Zool..

[2] Stevens J.R., Stephens D.W., Madden G.J., Bickel W.K. (2010). The adaptive nature of impulsivity. Impulsivity: The Behavioral and Neurological Science of Discounting.

[3] De Petrillo F., Ventricelli M., Ponsi G., Addessi E. (2015). Do tufted capuchin monkeys play the odds? Flexible risk preferences in *Sapajus* spp.. Anim. Cogn..

[4] Heilbronner S.R., Rosati A.G., Stevens J.R., Hare B., Hauser M.D. (2008). A fruit in the hand or two in the bush? Divergent risk preferences in chimpanzees and bonobos. Biol. Lett..

[5] De Petrillo F., Rosati A.G. (2021). Variation in primate decision-making under uncertainty and the roots of human economic behaviour. Philos. Trans. R. Soc. B.

[6] Rosati A.G. (2017). Foraging cognition: Reviving the ecological intelligence hypothesis. Trends Cogn. Sci..

[7] Eisenreich B.R., Hayden B.Y., Zimmermann J. (2019). Macaques are risk-averse in a freely moving foraging task. Sci. Rep..

[8] Farashahi S., Azab H., Hayden B.Y., Soltani A. (2018). On the flexibility of basic risk attitudes in monkeys. J. Neurosci..

[9] Ferrari-Toniolo S., Bujold P.M., Schultz W. (2019). Probability distortion depends on choice sequence in rhesus monkeys. J. Neurosci..

[10] Haux L.M., Engelmann J.M., Arslan R.C., Hertwig R., Herrmann E. (2023). Chimpanzee and human risk preferences show key similarities. Psychol. Sci..

[11] Haux L.M., Engelmann J.M., Herrmann E., Hertwig R. (2021). How chimpanzees decide in the face of social and nonsocial uncertainty. Anim. Behav..

[12] Roig A., Ferretti A., Gastaldi S., Meunier H., Addessi E. (2026). Tempted yet cautious: How described odds lower risk aversion in capuchins. Anim. Behav..

[13] Gilby I.C., Machanda Z.P., O’Malley R.C., Murray C.M., Lonsdorf E.V., Walker K., Mjungu D.C., Otali E., Muller M.N., Emery Thompson M. (2017). Predation by female chimpanzees: Toward an understanding of sex differences in meat acquisition in the last common ancestor of *Pan* and *Homo*. J. Hum. Evol..

[14] Ciacci F., Mayerhoff S., De Petrillo F., Gastaldi S., Brosnan S.F., Addessi E. (2023). State-dependent risky choices in primates: Variation in energy budget does not affect tufted capuchin monkeys’ (*Sapajus* spp.) risky choices. Am. J. Primatol..

[15] De Petrillo F., Rossi F., Gastaldi S., Addessi E. (2023). Do tufted capuchin monkeys, *Sapajus* spp., experience regret in decision making under risk?. Anim. Behav..

[16] Rivière J., Kurt A., Meunier H. (2019). Choice under risk of gain in tufted capuchin monkeys (*Sapajus apella*): A comparison with young children (*Homo sapiens*) and mangabey monkeys (*Cercocebus torquatus torquatus*). J. Neurosci. Psychol. Econ..

[17] Rosati A.G., Thompson M.E., Atencia R., Buckholtz J.W. (2023). Distinct developmental trajectories for risky and impulsive decision-making in chimpanzees. J. Exp. Psychol. Gen..

[18] Defoe I.N., Dubas J.S., Figner B., van Aken M.A.G. (2015). A meta-analysis on age differences in risky decision making: Adolescents versus children and adults. Psychol. Bull..

[19] De Petrillo F., Rosati A.G. (2019). Rhesus macaques use probabilities to predict future events. Evol. Hum. Behav..

[20] Haun D.B., Nawroth C., Call J. (2011). Great apes’ risk-taking strategies in a decision-making task. PLoS ONE.

[21] Broihanne M.H., Romain A., Call J., Thierry B., Wascher C.A., De Marco A., Verrier D., Dufour V. (2019). Monkeys (*Sapajus apella* and *Macaca tonkeana*) and great apes (*Gorilla gorilla*, *Pongo abelii*, *Pan paniscus*, and *Pan troglodytes*) play for the highest bid. J. Comp. Psychol..

[22] Pelé M., Broihanne M.H., Thierry B., Call J., Dufour V. (2014). To bet or not to bet? Decision-making under risk in non-human primates. J. Risk Uncertain..

[23] Teichroeb J.A., Smeltzer E.A., Mathur V., Anderson K.A., Fowler E.J., Adams F.V., Vasey E.N., Kumpan L.T., Stead S.M., Arseneau-Robar T.J.M. (2025). How can we apply decision-making theories to wild animal behavior? Predictions arising from dual process theory and Bayesian decision theory. Am. J. Primatol..

[24] Bosshard T.C., Mundry R., Fischer J. (2025). Ecological risk-taking across age in Barbary macaques. Anim. Behav..

[25] Gumert M.D. (2007). Payment for sex in a macaque mating market. Anim. Behav..

[26] van Noordwijk M.A., van Schaik C.P. (1987). Competition among female long-tailed macaques, *Macaca fascicularis*. Anim. Behav..

[27] Yeager C.P. (1996). Feeding ecology of the long-tailed macaque (*Macaca fascicularis*) in Kalimantan Tengah, Indonesia. Int. J. Primatol..

[28] Luncz L.V., Svensson M.S., Haslam M., Malaivijitnond S., Proffitt T., Gumert M.D. (2017). Technological response of wild macaques (*Macaca fascicularis*) to anthropogenic change. Int. J. Primatol..

[29] Keupp S., Abedin F., Jeanson L., Kade C., Kalbitz J., Titchener R., Mussweiler T., Bugnyar T., Fischer J. (2021). Performance-based social comparisons in humans and long-tailed macaques. Anim. Behav. Cogn..

[30] Massen J.J.M., van den Berg L.M., Spruijt B.M., Sterck E.H.M. (2012). Inequity aversion in relation to effort and relationship quality in long-tailed macaques (*Macaca fascicularis*). Am. J. Primatol..

[31] Pritchard A.J., Bliss-Moreau E., Balasubramaniam K.N., Capitanio J.P., Marty P.R., Kaburu S.S.K., Arlet M.E., Beisner B.A., McCowan B. (2024). Personality trait structures across three species of *Macaca*, using survey ratings of responses to conspecifics and humans. PLoS ONE.

[32] Sussman A.F., Ha J.C., Bentson K.L., Crockett C.M. (2013). Temperament in rhesus, long-tailed, and pigtailed macaques varies by species and sex. Am. J. Primatol..

[33] Hasan M.U., Widayati K.A., Tsuji Y., Rianti P. (2023). Feeding ecology of free-ranging long-tailed macaques in East Java, Indonesia: Relationship with human food availability. Primates.

[34] Leca J.-B., Gunst N., Gardiner M., Wandia I.N. (2021). Acquisition of object robbing and object/food bartering: A culturally maintained token economy in free-ranging long-tailed macaques. Philos. Trans. R. Soc. B.

[35] Bruchmann C., Stolla J., Titchener R., Fischer J., Keupp S. (2023). How does social context modulate risky decision-making in long-tailed macaques (*Macaca fascicularis*)?. OSF Preprints.

[36] Brotcorne F., Holzner A., Jorge-Sales L., Gunst N., Hambuckers A., Wandia I.N., Leca J.-B. (2020). Social influence on the expression of robbing and bartering behaviours in Balinese long-tailed macaques. Anim. Cogn..

[37] Fuentes A., Southern M., Suaryana K.G., Patterson J.D., Wallis J. (2005). Monkey forests and human landscapes: Is extensive sympatry sustainable for *Homo sapiens* and *Macaca fascicularis* on Bali?. Commensalism and Conflict: The Human–Primate Interface.

[38] Brotcorne F. (2014). Behavioral Ecology of Commensal Long-Tailed Macaque (*Macaca fascicularis*) Populations in Bali, Indonesia: Impact of Anthropic Factors. Ph.D. Thesis.

[39] Brotcorne F., Fuentes A., Wandia I.N., Beudels-Jamar R.C., Huynen M.C. (2015). Changes in activity patterns and intergroup relationships after a significant mortality event in commensal long-tailed macaques (*Macaca fascicularis*) in Bali, Indonesia. Int. J. Primatol..

[40] Heilbronner S.R., Hayden B.Y. (2013). Contextual factors explain risk-seeking preferences in rhesus monkeys. Front. Neurosci..

[41] Yamada H., Tymula A., Louie K., Glimcher P.W. (2013). Thirst-dependent risk preferences in monkeys identify a primitive form of wealth. Proc. Natl. Acad. Sci. USA.

[42] Morales J.C., Melnick D.J. (1998). Phylogenetic relationships of the macaques (*Cercopithecidae*: *Macaca*), as revealed by high resolution restriction site mapping of mitochondrial ribosomal genes. J. Hum. Evol..

[43] Thierry B. (2007). Unity in diversity: Lessons from macaque societies. Evol. Anthropol..

[44] Thierry B., Aureli F., Nunn C.L., Petit O., Abegg C., de Waal F.B.M. (2008). A comparative study of conflict resolution in macaques: Insights into the nature of trait covariation. Anim. Behav..

[45] Loyant L., Waller B.M., Micheletta J., Meunier H., Ballesta S., Joly M. (2023). Tolerant macaque species are less impulsive and reactive. Anim. Cogn..

[46] Pelé M., Dufour V., Micheletta J., Thierry B. (2010). Long-tailed macaques display unexpected waiting abilities in exchange tasks. Anim. Cogn..

[47] Stolla J.J., Keupp S. (2024). The effect of reward value on the performance of long-tailed macaques (*Macaca fascicularis*) in a delay-of-gratification exchange task. Primate Biol..

[48] Johnson K.L., Bixter M.T., Luhmann C.C. (2020). Delay discounting and risky choice: Meta-analytic evidence regarding single-process theories. Judgm. Decis. Mak..

[49] Keupp S., Grueneisen S., Warneken F., Ludvig E.A., Melis A.P. Relationship between delay discounting and risk preference in chimpanzees (*Pan troglodytes*) and humans. Proceedings of the Annual Meeting of the Cognitive Science Society.

[50] Hertwig R., Erev I. (2009). The description–experience gap in risky choice. Trends Cogn. Sci..

[51] Hertwig R., Barron G., Weber E.U., Erev I. (2004). Decisions from experience and the effect of rare events in risky choice. Psychol. Sci..

[52] Fairbanks L.A. (1993). Risk-taking by juvenile vervet monkeys. Behaviour.

[53] Bernstein I.S., Ehardt C.L. (1986). Modification of aggression through socialization and the special case of adult and adolescent male rhesus monkeys (*Macaca mulatta*). Am. J. Primatol..

[54] Ren S., Liu S., Sun W., Gao L., Ren L., Liu J., Zhang W., Xia D., Sun B., Li J. (2024). Consistent individual differences drive collective movements in a Tibetan macaque group. Animals.

[55] Key C., Ross C. (1999). Sex differences in energy expenditure in non-human primates. Proc. R. Soc. Lond. B.

[56] Gilby I.C., Wrangham R.W. (2007). Risk-prone hunting by chimpanzees (*Pan troglodytes schweinfurthii*) increases during periods of high diet quality. Behav. Ecol. Sociobiol..

[57] Rosati A.G., Hare B. (2013). Chimpanzees and bonobos exhibit emotional responses to decision outcomes. PLoS ONE.

[58] Blanchard T.C., Wilke A., Hayden B.Y. (2014). Hot-hand bias in rhesus monkeys. J. Exp. Psychol. Anim. Learn. Cogn..

[59] Strait C.E., Blanchard T.C., Hayden B.Y. (2014). Reward value comparison via mutual inhibition in ventromedial prefrontal cortex. Neuron.

[60] Xu E.R., Kralik J.D. (2014). Risky business: Rhesus monkeys exhibit persistent preferences for risky options. Front. Psychol..

[61] Keupp S., Grueneisen S., Ludvig E.A., Warneken F., Melis A.P. (2021). Reduced risk-seeking in chimpanzees in a zero-outcome game. Philos. Trans. R. Soc. B.

[62] Carter A.J., Marshall H.H., Heinsohn R., Cowlishaw G. (2012). Personality predicts the propensity for social learning in a wild primate. Proc. R. Soc. Lond. B Biol. Sci..

[63] Carter A.J., Marshall H.H., Cowlishaw G., Heinsohn R. (2013). How not to measure boldness: Novel object and antipredator responses are not the same in wild baboons. Anim. Behav..

[64] Damerius L.A., Forss S.I., Kosonen Z.K., Willems E.P., Burkart J.M., Call J., Galdikas B.M., Liebal K., Haun D.B., van Schaik C.P. (2017). Orientation toward humans predicts cognitive performance in orang-utans. Sci. Rep..

[65] Kulik L., Amici F., Langos D., Widdig A. (2015). Sex differences in the development of social relationships in rhesus macaques (*Macaca mulatta*). Int. J. Primatol..

[66] Amici F., Kulik L., Langos D., Widdig A. (2019). Growing into adulthood—A review on sex differences in the development of sociality across macaques. Behav. Ecol. Sociobiol..

